# Libel Suit

**Published:** 1856-11

**Authors:** 


*Libel Suit.*—Dr. Goadby, one of the editors of the Detroit Medical Independent, has commenced a suit for libel against Dr. Z. Pitcher, one of the editors of the Peninsular Journal, and President of the American Medical Association.

